# Regulatory mechanism of the SenX3-RegX3 two-component system in *Mycobacterium smegmatis*: Roles of PhoU in sensing inorganic phosphate levels

**DOI:** 10.1016/j.jbc.2025.110435

**Published:** 2025-06-28

**Authors:** Youjin Seung, Ha-Eun Baik, Eun-Jin Park, Jeong-Il Oh

**Affiliations:** 1 Department of Integrated Biological Science, Pusan National University, Busan, South Korea; 2 Department of Medical Science, College of Medicine, Chungnam National University, Daejeon, South Korea; 3 Microbiological Resource Research Institute, Pusan National University, Busan, South Korea

**Keywords:** gene regulation, histidine kinase, *Mycobacterium*;, phosphate, response regulator, RegX3, SenX3, two-component system

## Abstract

The SenX3-RegX3 two-component system (TCS) in mycobacteria, consisting of the SenX3 histidine kinase and the RegX3 response regulator, regulates gene expression related to inorganic phosphate (Pi) acquisition in response to environmental Pi availability. In this study, we investigated how SenX3 senses environmental Pi levels and examined the role of the two PhoU paralogs, PhoU1 and PhoU2, in Pi sensing in association with the SenX3-RegX3 TCS in *Mycobacterium smegmatis*. Our findings revealed that while membrane localization of SenX3 is not required for its sensory function, its Per-ARNT-Sim domain is essential for Pi sensing. Contrary to previously proposed models, the Pst system, a high-affinity Pi transporter, does not directly participate in Pi sensing or signal transduction through the SenX3-RegX3 TCS. Instead, the PhoU paralogs likely sense intracellular Pi levels independent of the Pst system and modulate the balance between the kinase and phosphatase activities of SenX3. Using *in vitro* kinase and phosphatase assays, we demonstrated that purified PhoU1 shifts SenX3 activity toward its phosphatase function in the presence of Pi, thereby promoting the dephosphorylation of phosphorylated RegX3. We further confirmed that PhoU1 and PhoU2 are functionally redundant in *M. smegmatis*. Finally, we found that the expression of *phoU1* and *phoU2* is induced under Pi-deficient conditions: *phoU1* upregulation is mediated by the SenX3-RegX3 TCS, whereas *phoU2* expression is induced by SigF, an alternative sigma factor.

The intracellular pool of inorganic phosphate (Pi) is maintained at a relatively constant level through precise regulation of Pi acquisition, as Pi is essential for vital metabolic processes, including central dogma processes and energy metabolism (1). In *Escherichia coli*, the PhoRB two-component system (TCS), associated with the high-affinity Pi transporter Pst *via* PhoU, regulates genes involved in Pi acquisition and scavenging in response to environmental Pi availability (1, 2). PhoR is a histidine kinase (HK), while PhoB is its cognate response regulator (RR) (3). PhoR is a membrane protein with two transmembrane (TM) helices in its N-terminal domain (1, 4, 5, 6). In the remaining portion of PhoR, it contains a Per-ARNT-Sim (PAS) domain, along with a kinase domain consisting of the dimerization and histidine phosphorylation (DHp) and catalytic ATP-binding (CA) domains (1, 7, 8). The PAS domains in various sensory proteins have been suggested to function as sensory modules that detect oxygen, redox potential, light, and a variety of other stimuli (9). The PhoR HK has both kinase and phosphatase activities. Under Pi-deficient conditions, PhoR autophosphorylates a conserved histidine residue in the DHp domain, transferring the phosphoryl group to an aspartate residue in the receiver domain of PhoB, thereby activating its transcriptional activity. Conversely, under Pi-replete conditions, PhoR shifts its catalytic equilibrium toward phosphatase activity, dephosphorylating PhoB to suppress its function (1, 2, 3, 7, 10).

As a functionally homologous regulatory system, the SenX3-RegX3 TCS in mycobacteria has been suggested to regulate genes for Pi acquisition and scavenging in response to changes in environmental Pi levels (11, 12). The SenX3-RegX3 TCS is implicated in virulence, persister formation, survival under oxidative stress, and membrane vesicle biogenesis (13, 14, 15, 16, 17, 18, 19, 20). The SenX3 HK, an ortholog of PhoR, has a TM domain at its N terminus (11). Intriguingly, the PAS domain of SenX3 was demonstrated to contain a *b*-type heme, enabling the HK to sense diatomic gases such as O_2_, NO, and CO (14). During Pi deprivation, the SenX3 HK phosphorylates the RegX3 RR, which in turn activates the transcription of its regulon, including the *pstSCAB* operon encoding the high-affinity Pi transporter (Pst system) and the *phoA* gene encoding alkaline phosphatase (11, 12).

The Pst system consists of four types of polypeptides. PstS is a periplasmic substrate-binding protein, while PstA and PstC form a membrane-spanning channel. PstB functions as an ATPase (21, 22, 23, 24). The expression of the *pstSCAB* operon is induced under Pi-deficient conditions as part of the PhoB and RegX3 regulons in *E. coli* and mycobacteria, respectively (1, 11, 12).

The PhoU homologs have been suggested to participate in Pi signal transduction together with the Pst transport system and PhoR (SenX3) (2, 17, 25, 26, 27). In *E. coli* grown under Pi-replete conditions, PhoU has been shown to transmit an inhibitory signal generated from the Pst system to the PhoR HK, shifting the equilibrium of kinase and phosphatase activities toward the phosphatase-dominant mode by directly interacting with both the PAS domain of PhoR and the PstB subunit of the Pst transporter (1, 2). According to this model, inactivation of the Pst transporter or PhoU by null mutations results in the constitutive expression of the PhoB regulon, regardless of environmental Pi availability (2, 28, 29, 30). Alternatively, several studies have suggested that intracellular Pi levels, rather than extracellular Pi levels, are sensed and influence PhoR kinase/phosphatase activity, although the molecular mechanism underlying this model has yet to be elucidated (31, 32, 33).

*Mycobacterium tuberculosis* and *Mycobacterium smegmatis* have two copies of *phoU* homologs: *phoY1* (*Rv3301c*) and *phoY2* (*Rv0821c*) in *M. tuberculosis* and *phoU1* (*MSMEG_5776*) and *phoU2* (*MSMEG_1605*) in *M. smegmatis* (18, 25). In *M. smegmatis*, the *phoU1* gene is located adjacent to the *pstSCAB* operon, whereas the *phoU2* gene is located in a genomic region separate from this locus (25). The PhoY1 and PhoY2 paralogs have been shown to function redundantly to each other in inhibiting the SenX3-RegX3 TCS under P_i_-replete conditions in *M. tuberculosis*, similar to the redundancy observed between the PhoU1 and the PhoU2 paralogs in *M. smegmatis* (18, 25). In both *M. tuberculosis* and *M. smegmatis*, inactivation of PhoU homologs has been shown to increase susceptibility to rifampicin (18, 25). Conversely, overexpression of *phoU1* or *phoU2* has been observed to increase tolerance to rifampicin in *M. smegmatis* (34).

In this study, we provide multiple lines of evidence indicating that the Pst system does not directly participate in Pi sensing or signal transduction through the SenX3-RegX3 TCS. Instead, the PhoU paralogs appear to sense intracellular Pi levels and regulate the equilibrium of SenX3 kinase and phosphatase activities.

## Results

### The PAS domain, but not membrane localization, is essential for the Pi-sensing function of SenX3

**Figure 1:**
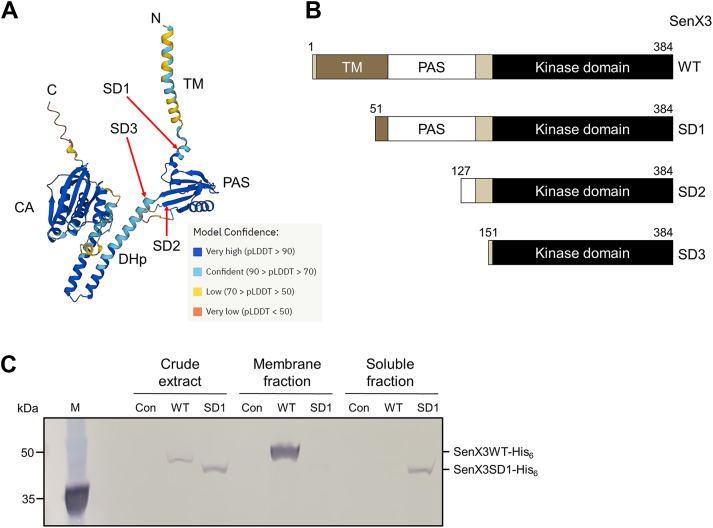
**Schematic diagrams illustrating the domain structures of the WT and serially deleted forms of SenX3, as well as the effect of removing the putative TM domain on its subcellular localization.***A*, predicted three-dimensional structure of SenX3 by AlphaFold. The per-residue confidence score (pLDDT) reflects prediction confidence, with 100 representing the highest confidence. The polypeptide is color-coded as follows: *orange* (pLDDT < 50), *yellow* (50 < pLDDT < 70), *light blue* (70 < pLDDT < 90), and *dark blue* (pLDDT > 90). *Arrows* indicate the positions of the first amino acids in the N-terminally truncated forms of SenX3. *B*, schematic representation of the WT and serially deleted forms (SD1, SD2, and SD3) of SenX3. The numbers above the diagrams indicate the first and last amino acids of the N-terminally truncated forms of SenX3. *C*, His_6_-tagged WT and SD1 forms of SenX3 were overexpressed in the Δ*senX3* strain of *M*. *smegmatis* containing pMHSenX3WT and pMHSenX3SD1, respectively. The Δ*senX3* strain with the empty vector pMH201 served as a control (Con). Cultures were grown aerobically in 7H9-glucose medium supplemented with 0.2% (w/v) acetamide until reaching an OD_600_ of 0.45 to 0.5. Western blot analysis was performed using 20 μg of protein from cell-free crude extracts, membrane fractions, and soluble fractions to detect the expressed His_6_-tagged WT and SD1 forms of SenX3. Membrane and soluble fractions were obtained from crude extracts by ultracentrifugation at 100,000*g* for 90 min. Abbreviations: TM, transmembrane domain; CA, catalytic ATP-binding domain; DHp, dimerization and histidine-containing phosphotransfer domain; PAS, Per-ARNT-Sim domain.

The SenX3 HK is predicted to be a membrane-associated HK containing a PAS domain located between the membrane-spanning α-helix and the kinase domain (20). Its kinase domain exhibits a prototypical structure, consisting of the DHp and CA domains (8) (Fig. 1*A*). It is possible that the SenX3 HK forms a membrane-associated signaling complex with the Pst Pi transporter and PhoU, similar to the PhoR HK in *E. coli*, where the Pst transporter functions as a Pi sensor and PhoU serves as a signal transmitter. To examine whether membrane association and the presence of the PAS domain are essential for the Pi-sensing and signaling function of SenX3, we constructed several plasmids to express N-terminally deleted His_6_-tagged SenX3 proteins in a *senX3* mutant strain (Δ*senX3*) (Fig. 1*B*). SenX3SD1 is a truncated form of SenX3 lacking the TM domain, while SenX3SD2 and SenX3SD3 lack both the TM and the PAS domains.

First, we assessed the role of the predicted TM domain in the membrane localization of SenX3. When the WT form of SenX3 was overexpressed in the Δ*senX3* mutant carrying pMHSenX3WT, the expressed His_6_-tagged WT SenX3 was detected in both crude extracts and membrane fractions but was absent from soluble fractions (Fig. 1*C*). In contrast, when SenX3SD1 was overexpressed in the Δ*senX3* mutant with pMHSenX3SD1, the His_6_-tagged SenX3SD1 was detected in both crude extracts and soluble fractions but not in membrane fractions. As expected, no His_6_-tagged protein band was observed in the negative control strain (Δ*senX3* carrying the empty vector pMH201). These results indicate that SenX3SD1, which lacks the TM domain, does not associate with the membrane and instead remains as a cytoplasmic soluble protein.

**Figure 2:**
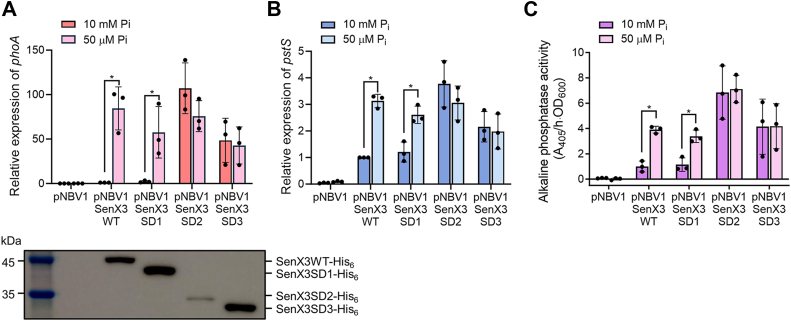
**Effects of serial deletions of SenX3 on its Pi-sensing capability.***A*, expression levels of *phoA* in the Δ*senX3* strains of *M. smegmatis* expressing either the WT or serially deleted forms (SD1, SD2, and SD3) of SenX3. *B*, expression levels of *pstS* in the Δ*senX3* strains expressing either the WT or serially deleted forms of SenX3. The Δ*senX3* mutant was complemented by introducing pNBV1 derivatives carrying the WT or mutant forms of *senX3* under the control of the *senX3* promoter (pNBV1SenX3WT, pNBV1SenX3SD1, pNBV1SenX3SD2, and pNBV1SenX3SD3. The Δ*senX3* mutant with the empty vector pNBV1 served as a control. The expression levels of *phoA* and *pstS* were quantitatively determined by qRT-PCR and normalized to that of *sigA*. The expression levels of *phoA* and *pstS* in the Δ*senX3* mutant with pNBV1SenX3WT grown in the presence of 10 mM Pi were set to 1, and the relative values for the others were expressed accordingly. Western blot analysis was performed to verify the expression of His_6_-tagged WT and serially deleted forms of SenX3. Cell-free crude extracts (20 μg) were separated by SDS-PAGE and analyzed by Western blotting using a His-tag antibody to detect His_6_-tagged proteins and an Hsp65 antibody to detect GroEL. The protein level of GroEL was used as a loading control. *C*, alkaline phosphatase assay. The activity of expressed PhoA was measured in the Δ*senX3* strains harboring pNBV1SenX3WT, pNBV1SenX3SD1, pNBV1SenX3SD2, or pNBV1SenX3SD3. The Δ*senX3* mutant with pNBV1 served as a control. The strains used in the experiments (panels A, B, and C) were grown aerobically to an OD_600_ of 0.45 to 0.5 in MOPS-glucose medium supplemented with either 10 mM or 50 μM K_2_HPO_4_ as the sole phosphate source. All values were determined from three biological replicates, and error bars indicate the standard deviations. ∗*p* < 0.05.

Next, we examined the Pi-sensing function of the serially deleted SenX3 variants by measuring the expression levels of *phoA* and *pstS* in Δ*senX3* strains expressing the SenX3 variants grown under Pi-replete (10 mM) and Pi-deficient (50 μM) conditions. Additionally, alkaline phosphatase activity was measured in *M. smegmatis* strains. Since the expression of *phoA* and *pstS* is upregulated in *M. smegmatis* by RegX3 under Pi-deficient conditions (11), these genes served as reporter genes to evaluate the sensory function of SenX3. We used the plasmids pNBV1SenX3WT, pNBV1SenX3SD1, pNBV1SenX3SD2, and pNBV1SenX3SD3 to express the His_6_-tagged WT and serially deleted forms of SenX3 from the original *senX3* promoter in the Δ*senX3* strain. As shown in Figure 2*A*, *phoA* was not expressed in the negative control Δ*senX3* strain carrying the empty vector pNBV1. In contrast, *phoA* expression was strongly induced in the Δ*senX3* mutant carrying pNBV1SenX3SD1 under Pi-deficient conditions compared to Pi-replete conditions, similar to the mutant with pNBV1SenX3WT expressing the WT form of SenX3. However, in the Δ*senX3* mutant carrying either pNBV1SenX3SD2 or pNBV1SenX3SD3, *phoA* expression remained derepressed even under Pi-replete conditions, indicating a loss of regulatory control over *phoA* expression. A similar pattern was observed for *pstS* expression and alkaline phosphatase activity (Fig. 2, *B* and *C*). The expression of the WT and mutant forms of SenX3 in the Δ*senX3* mutant was confirmed by Western blot analysis using an anti-His-tag monoclonal antibody (Fig. 2*A*). Taken together, these results indicate that the SenX3 HK retains its Pi-sensing ability even without membrane association, provided that the PAS domain remains intact.

### Changes in extracellular and intracellular Pi levels affect SenX3 function in the absence of the Pst transporter system

**Figure 3:**
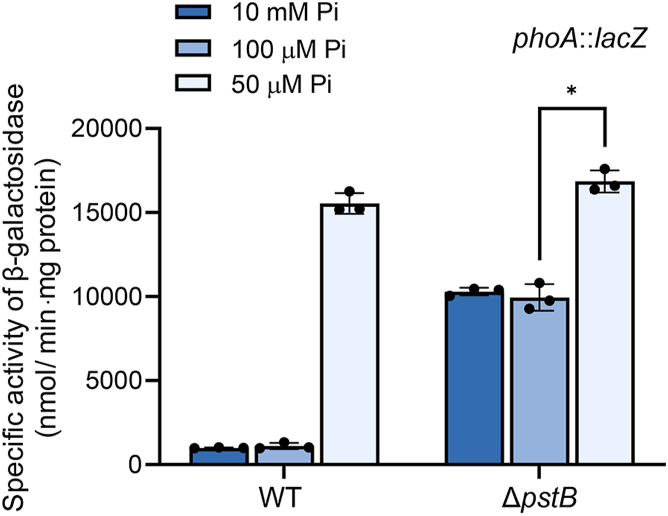
**Expression levels of *phoA* in the WT and Δ*pstB* strains of *M. smegmatis* grown under various Pi conditions.** The expression levels of *phoA* were determined using the *phoA*::*lacZ* transcriptional fusion plasmid pNCphoA. Strains were grown aerobically to an OD_600_ of 0.45 to 0.5 in MOPS-glucose medium supplemented with 10 mM, 100 μM, or 50 μM K_2_HPO_4_ as the sole phosphate source. β-galactosidase activity was measured in cell-free crude extracts. All values were derived from three biological replicates, and error bars indicate the standard deviations. ∗*p* < 0.05.

We demonstrated that the membrane association of SenX3 is not necessary for its Pi-sensing function in *M. smegmatis*, which contradicts the Pi-sensing model suggesting the formation of a membrane-associated Pst-PhoU-SenX3 signaling complex. We assessed whether the Pi-sensing function of SenX3 is completely lost in *M. smegmatis* in the absence of the Pst transporter. To examine this, we measured the expression level of *phoA* in the Δ*pstB* mutant grown at varying Pi concentrations. As a control, the WT strain of *M. smegmatis* was included in the experiment. As shown in Figure 3, *phoA* expression in the WT strain was significantly induced at 50 μM Pi compared to its expression at 10 mM and 100 μM Pi. *phoA* expression was derepressed in the Δ*pstB* mutant grown at 10 mM and 100 μM Pi relative to that observed in the WT strain grown at the same Pi concentrations. It is noteworthy that *phoA* expression was still induced in the Δ*pstB* mutant grown at 50 μM Pi compared to its expression in the mutant grown at 10 mM and 100 μM Pi. This result suggests that the SenX3 HK still retains its Pi-sensing ability in the absence of the Pst transporter. The derepression of *phoA* in the Δ*pstB* mutant relative to the WT strain is likely due to reduced intracellular Pi levels resulting from the absence of the major Pi transporter, Pst.

**Figure 4:**
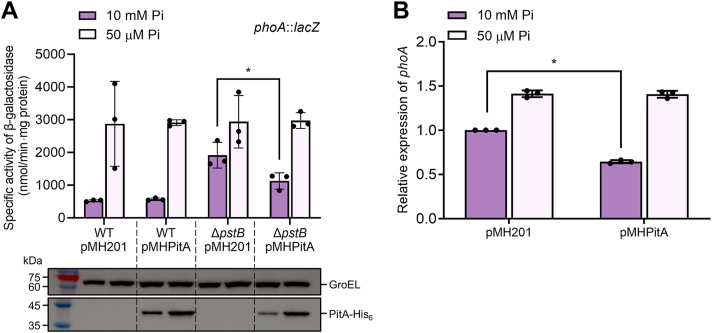
**Effects of *pitA* overexpression on *phoA* expression in the WT and Δ*pstB* strains of *M. smegmatis*.***A*, determination of *phoA* expression using the *phoA*::*lacZ* transcriptional fusion plasmid pNCphoA. The *pitA* gene was overexpressed in *M. smegmatis* using the pMH201-derived plasmid pMHPitA. The effect of *pitA* overexpression on *phoA* expression was assessed by measuring β-galactosidase activity in the WT and Δ*pstB* strains carrying both pNCphoA and pMHPitA. As negative controls, *M. smegmatis* strains carrying both pNCphoA and the empty vector pMH201 were included. Western blot analysis was performed to confirm the expression of His_6_-tagged PitA. Cell-free crude extracts (6.5 μg) were separated by SDS-PAGE and analyzed by Western blot analysis using a His-tag antibody. *B*, validation of *phoA* expression by qRT-PCR. *phoA* transcript levels were quantitatively measured in Δ*pstB* strains harboring either pMH201 or pMHPitA, with normalization to *sigA* expression. The expression level of *phoA* in the Δ*pstB* mutant carrying pMH201 grown in the presence of 10 mM Pi was set to 1, and the relative values were calculated for the other conditions. All strains were grown aerobically to an OD_600_ of 0.45 to 0.5 in MOPS-glucose medium supplemented with either 10 mM or 50 μM K_2_HPO_4_ as the sole phosphate source, along with 0.1% (w/v) of acetamide. All values presented were derived from three biological replicates, and error bars indicate the standard deviations. ∗*p* < 0.05.

We then examined whether changes in intracellular Pi levels affect the function of the SenX3-RegX3 TCS in *M. smegmatis* in the absence of the Pst transporter (Fig. 4). To alter intracellular Pi levels in the Δ*pstB* mutant, the low-affinity Pi transporter PitA, which consists of a single polypeptide (35), was overexpressed in the mutant using the pMH201-derived pMHPitA, where the *pitA* gene is under the control of an acetamide-inducible promoter. As a control, the WT strain was included in the experiment. The function of SenX3 was determined by measuring the expression level of *phoA* using pNCphoA. As shown in Figure 4*A*, *phoA* expression was induced in the WT strain carrying the empty vector pMH201 under Pi-deficient conditions (50 μM Pi) compared to Pi-replete conditions (10 mM Pi). The Δ*pstB* mutant with pMH201 grown at 10 mM Pi exhibited derepressed *phoA* expression relative to the WT strain grown under the same conditions. Furthermore, the Δ*pstB* strain carrying pMH201 grown at 50 μM Pi showed increased *phoA* expression compared to the same strain grown at 10 mM Pi. These observations are consistent with the results shown in Figure 3. Interestingly, overexpression of *pitA* in the Δ*pstB* mutant with pMHPitA led to a decrease in *phoA* expression compared to the mutant strain with pMH201 when both were grown at 10 mM Pi. This effect of *pitA* overexpression in the Δ*pstB* mutant was confirmed by qRT-PCR (Fig. 4*B*). In contrast, no overexpression effect of *pitA* was observed in the Δ*pstB* mutant grown at 50 μM Pi or in the WT strain grown under either Pi condition (Fig. 4*A*). The overexpression of *pitA* in *M. smegmatis* strains was verified by Western blot analysis (Fig. 4*A*). The overexpression effect of *pitA* observed only in the Δ*pstB* mutant grown at 10 mM Pi can be explained as follows: Under 10 mM Pi conditions, intracellular Pi levels in the Δ*pstB* mutant are likely lower than those of the WT strain, leading to increased *phoA* expression. The overexpression of *pitA* in the Δ*pstB* mutant likely partially restores intracellular Pi levels, thereby reducing *phoA* expression. In the WT strain grown at 10 mM Pi, intracellular Pi levels are presumably already sufficient to repress *phoA* expression. Thus, a further increase in Pi levels by *pitA* overexpression does not have an additional effect on *phoA* expression. Under 50 μM Pi conditions, the low-affinity Pi transporter PitA likely cannot function properly, thereby preventing the overexpression effect of *pitA* in both the WT and Δ*pstB* strains.

### The two PhoU paralogs are functionally redundant, and their genes are upregulated under Pi-deficient conditions

**Figure 5:**
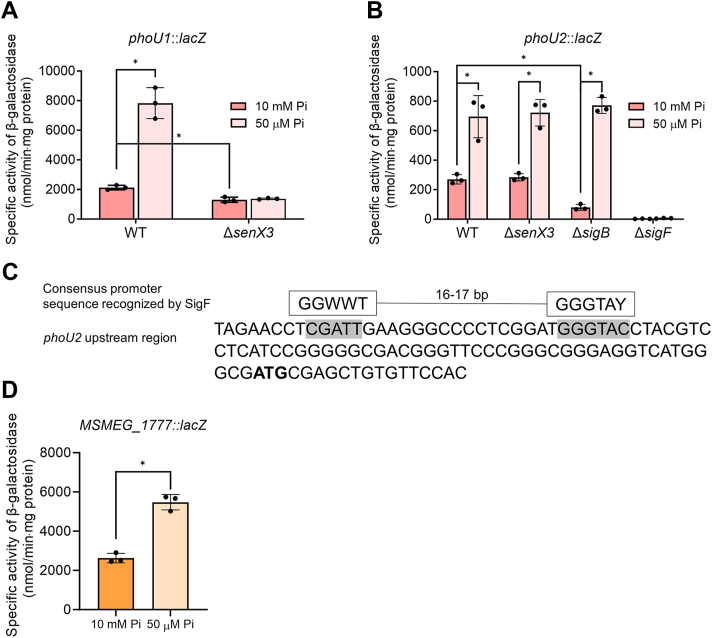
**Induction of *phoU1* and *phoU2* expression in *M. smegmatis* under Pi-deficient conditions and identification of involved regulatory systems.***A*, *phoU1* expression levels in the WT and Δ*senX3* strains of *M. smegmatis*. *B*, *phoU2* expression levels in the WT, Δ*senX3*, Δ*sigB*, and Δ*sigF* strains of *M. smegmatis*. *C*, upstream regions of *phoU2*. The start codon is bolded, and the predicted promoter region is highlighted in *gray*. The suggested SigF-recognizing promoter (49) is presented above the *phoU2* sequence. *D*, expression levels of *MSMEG_1777* in the WT strain of *M. smegmatis*. Expression levels of *phoU1*, *phoU2*, and *MSMEG_1777* were determined in WT and mutant strains harboring pNCⅡphoU1, pNCⅡphoU2, and pNCII1777, respectively. Strains were grown aerobically to an OD_600_ of 0.45 to 0.5 in MOPS-glucose medium supplemented with 10 mM or 50 μM K_2_HPO_4_ as the sole phosphate source. All values presented were derived from three biological replicates, and error bars represent the standard deviations. ∗*p* < 0.05.

Previously, it was demonstrated that PhoU1 and PhoU2 function redundantly to inhibit the SenX3-RegX3 TCS in *M. smegmatis* under Pi-replete conditions, similar to the role of PhoY1 and PhoY2 in *M. tuberculosis* (18, 25). We constructed *phoU1* and *phoU2* single mutants, as well as a *phoU1phoU2* double mutant of *M. smegmatis*. We confirmed that full derepression of *phoA* under Pi-replete conditions (10 mM Pi) occurred only in the *phoU1phoU2* double mutant, but not in the single mutants (data not shown). Notably, the *phoU1phoU2* double mutant could be obtained only when mutant selection on sucrose plates was performed under Pi-deficient conditions (50 μM Pi), supporting the previous suggestion that the growth of *M. smegmatis* might be severely compromised by phosphate toxicity when both *phoU1* and *phoU2* are inactivated (25). The relative expression levels of *phoU1* and *phoU2* in *M. smegmatis* were extrapolated from the reads per kilo base pair per million mapped reads (RPKM) values obtained from RNA sequencing analysis of the WT strain of *M. smegmatis* that was aerobically grown to an OD_600_ of 0.45 to 0.5 in 7H9-glucose medium (25 mM Pi) (NCBI GEO accession number GSE155251) (36). The RPKM values of *phoU1* and *phoU2* indicate that the transcript level of *phoU1* is 3.6-fold higher than that of *phoU2* in the WT strain of *M. smegmatis* grown under Pi-replete conditions. We next examined whether the expression of *phoU1* and *phoU2* is regulated in response to Pi deficiency. The expression levels of *phoU1* and *phoU2* were measured in *M. smegmatis* strains using the *lacZ* translational fusion plasmids pNCIIphoU1 and pNCIIphoU2, respectively. As shown in Figure 5, *A* and *B*, the expression levels of *phoU1* and *phoU2* in the WT strain were increased by 3.7- and 2.6-fold, respectively, under Pi-deficient conditions compared to Pi-replete conditions. Since the SenX3-RegX3 TCS is involved in inducing gene expression under Pi-deficient conditions, we investigated whether it is responsible for the induction of *phoU1* and *phoU2* expression under Pi-deficient conditions. When grown under Pi-replete conditions, the Δ*senX3* mutant exhibited lower *phoU1* expression levels than the WT strain, and *phoU1* expression was not induced in the Δ*senX3* mutant grown under Pi-deficient conditions, unlike in the WT strain (Fig. 5*A*). In contrast, the inactivation of *senX3* did not affect the upregulation of *phoU2* under Pi-deficient conditions (Fig. 5*B*). We previously reported that *phoU2* expression was compromised in the Δ*sigB* mutant, in which the *sigB* gene encoding an alternative sigma factor is deleted, when the mutant was grown in 7H9-glucose medium (25 mM Pi) (34). Consistent with our previous report, *phoU2* expression was reduced in the Δ*sigB* mutant relative to the WT strain when both strains were grown under Pi-replete conditions. However, the expression level of *phoU2* in the Δ*sigB* mutant was induced to the level observed in the WT and Δ*senX3* strains when grown under Pi-deficient conditions, indicating that SigB and the SenX3-RegX3 TCS are not involved in the induction of *phoU2* expression under Pi-deficient conditions. A detailed examination of the upstream region of *phoU2* allowed us to identify a well-conserved SigF-recognizing promoter sequence (Fig. 5*C*). We found that *phoU2* expression was abolished in the Δ*sigF* mutant grown under both Pi-replete and Pi-deficient conditions (Fig. 5*B*), indicating that transcription of *phoU2* is directed by SigF-associated RNA polymerase. To ascertain whether SigF is responsible for the induction of *phoU2* expression under Pi-deficient conditions, we determined the expression level of *MSMEG_1777*, which belongs to the SigF regulon (36, 37), in the WT strain of *M. smegmatis* grown under Pi-replete and Pi-deficient conditions (Fig. 5*D*). Similar to *phoU2*, the expression level of *MSMEG_1777* was increased by 2.1-fold under Pi-deficient conditions compared to Pi-replete conditions, suggesting that the induction of *phoU2* expression in *M. smegmatis* grown under Pi-deficient conditions is mediated by SigF.

### PhoU alone is sufficient to regulate SenX3’s function in phosphorylating and dephosphorylating RegX3

**Figure 6:**
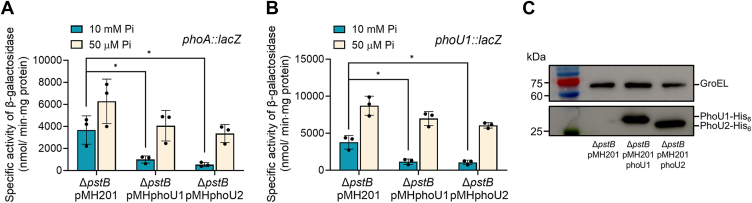
**Effects of *phoU1* or *phoU2* overexpression on *phoA* and *phoU1* expression in the Δ*pstB* mutant of *M. smegmatis*.***phoU1 and phoU2* were overexpressed in the Δ*pstB* strain using the pMH201-derived pMHphoU1 and pMHphoU2, respectively. *A*, effect of *phoU1* or *phoU2* overexpression on *phoA* expression. This was assessed using Δ*pstB* strains carrying pNCphoA and either pMHphoU1 or pMHphoU2. *B*, effect of *phoU1* or *phoU2* overexpression on *phoU1* expression. β-galactosidase activity was measured in Δ*pstB* strains carrying pNCIIphoU1 along with either pMHphoU1 or pMHphoU2. As negative controls, we included Δ*pstB* strains harboring pNCphoA plus the empty vector pMH201, or pNCIIphoU1 plus pMH201. All strains were grown aerobically to an OD_600_ of 0.45 to 0.5 in MOPS-glucose medium supplemented with either 10 mM K_2_HPO_4_ or 50 μM K_2_HPO_4_ as the sole phosphate source, along with 0.1% (w/v) of acetamide. All values were derived from three biological replicates, and error bars indicate the standard deviations. ∗*p* < 0.05. *C*, Western blot analysis confirming the expression of His_6_-tagged PhoU1 and PhoU2. *M. smegmatis* cultures were grown aerobically in 7H9-glucose medium supplemented with 0.1% (w/v) acetamide until reaching an OD_600_ of 0.45 to 0.5. 10 μg of cell-free crude extracts were resolved by SDS-PAGE and subsequently probed with a His-tag antibody.

To determine whether the PhoU paralogs can exert an inhibitory effect on the SenX3-RegX3 TCS in the absence of the Pst system, we examined the effect of *phoU1* and *phoU2* overexpression on the expression of *phoA* and *phoU1* in the Δ*pstB* mutant of *M. smegmatis* grown under Pi-replete and Pi-deficient conditions (Fig. 6). The overexpression of *phoU1* and *phoU2* in the Δ*pstB* mutant carrying pMHphoU1 and pMHphoU2, respectively, resulted in a decrease in *phoA* expression, particularly under Pi-replete conditions, compared to its expression level in the Δ*pstB* mutant with pMH201 (Fig. 6*A*). A similar effect of *phoU1* and *phoU2* overexpression on *phoU1* expression in the Δ*pstB* mutant was also observed (Fig. 6*B*). Since the inhibitory effect of *phoU1* and *phoU2* overexpression on both *phoA* and *phoU1* expression was more pronounced under Pi-replete conditions than under Pi-deficient conditions, the Pi-sensing function of the SenX3-RegX3 TCS appeared to be at least partially restored in the Δ*pstB* mutant carrying pMHphoU1 or pMHphoU2 compared to the Δ*pstB* mutant with pMH201, where Pi sensing is nearly dysfunctional. Importantly, this finding also suggests that the ability of PhoU to inhibit the SenX3-RegX3 TCS is influenced by Pi availability even in the absence of the Pst system. The overexpression of His_6_-tagged PhoU1 and PhoU2 in the Δ*pstB* mutant carrying pMHphoU1 and pMHphoU2, respectively, was verified by Western blot analysis using a His-tag antibody (Fig. 6*C*). Taken together, these results suggest that, in the absence of the Pst transport system, PhoU alone appears to regulate the function of the SenX3-RegX3 TCS in response to Pi availability, provided that cellular PhoU levels are adequate.

**Figure 7:**
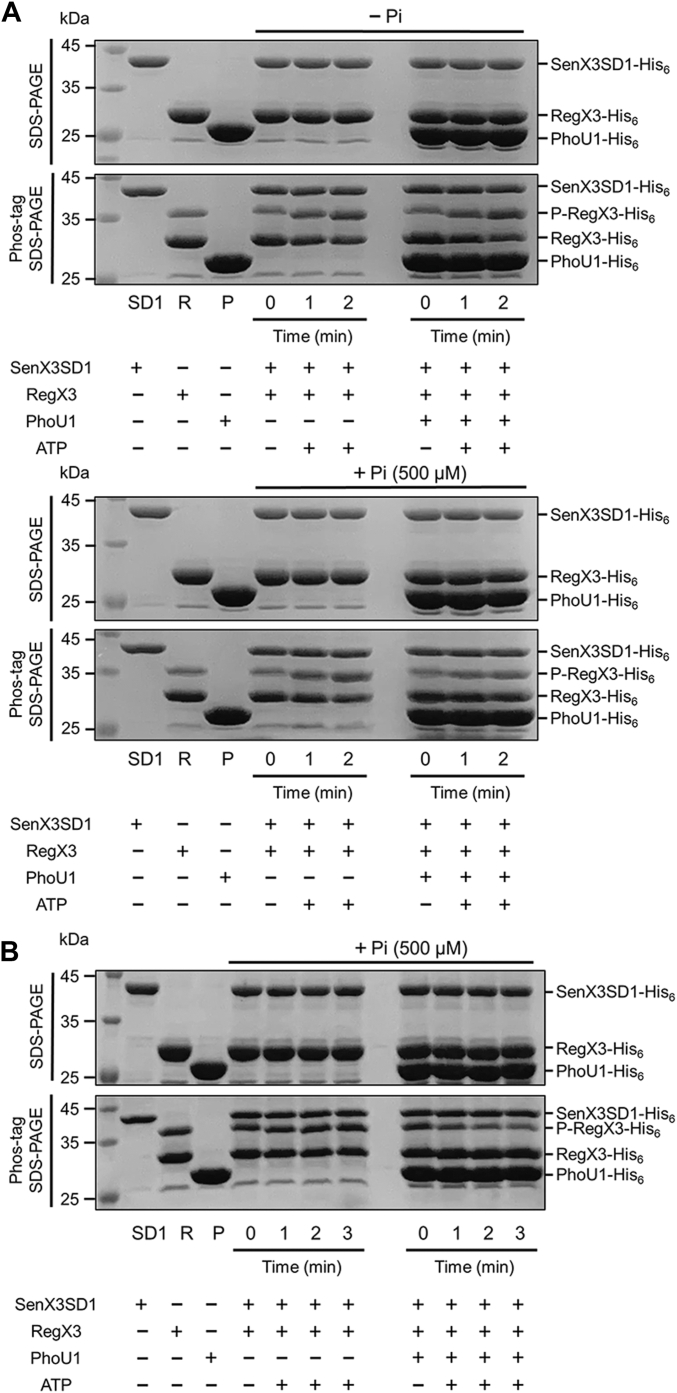
***In vitro* demonstration of PhoU1 and Pi-dependent modulation of SenX3 activity towards phosphatase function.***A*, effect of PhoU1 and Pi on SenX3 kinase activity. A total of 46 pmol of purified SenX3SD1 and 150 pmol of purified RegX3 were mixed in a reaction buffer [300 mM Tris-HCl (pH 8.0); 50 mM KCl; 10 mM MgCl_2_]. Where indicated, 275 pmol of purified PhoU1 and 500 μM Pi were included in the reaction mixture. The RegX3 substrate used was less phosphorylated RegX3, which was purified from *E. coli* grown to the mid-exponential phase. *B*, effect of PhoU1 on SenX3 phosphatase activity. Reaction mixtures contained 39 pmol of purified SenX3SD1 and 144 pmol of purified RegX3 in a reaction buffer [300 mM Tris-HCl (pH 8.0); 50 mM KCl; 10 mM MgCl_2_] with 500 μM Pi. To assess PhoU1’s effect, 200 pmol of purified PhoU1 was added to the reaction mixture. More phosphorylated RegX3, which was purified from *E. coli* grown to the late-exponential phase, was used. The preparation of the reaction mixtures was carried out on ice. Reactions were initiated by adding ATP to a final concentration of 1 mM and incubated for two or 3 min at 30 °C. The time point before ATP addition was designated as 0 min. Reactions were stopped by adding 3× loading buffer at the indicated time points. The reaction mixtures were then analyzed using both SDS-PAGE and Phos-tag SDS-PAGE. The gels were stained with CBB, and the bands corresponding to SenX3SD1, RegX3, phosphorylated RegX3 (P-RegX3), and PhoU1 are indicated by *arrows*. Lanes: SD1, SenX3 SD1; R, RegX3; P, PhoU1.

We performed *in vitro* kinase and phosphatase assays using purified SenX3SD1, RegX3, and PhoU1 to determine whether PhoU1 alone, in the absence of the Pst system, can regulate SenX3 activity in phosphorylating and dephosphorylating RegX3 (Fig. 7). We selected the SD1 form of SenX3 for this experiment because SenX3SD1 was shown to retain Pi-sensing function like the WT form of SenX3 and to be a soluble protein (Figs. 1 and 2). Unexpectedly, we found that the RegX3 protein purified from *E. coli* after overexpression of its gene was partially phosphorylated. Moreover, the fraction of phosphorylated RegX3 was found to be higher in the protein purified from *E. coli* grown to the late exponential phase compared to the mid-exponential phase (data not shown). We first examined the effect of PhoU1 and Pi on SenX3 activity in phosphorylating RegX3 (Fig. 7*A*). In this experiment, we used a less phosphorylated form of SenX3 to better observe its kinase activity. In the absence of Pi in the reaction mixture (-Pi), SenX3SD1 phosphorylated RegX3 at a similar rate, regardless of the presence or absence of PhoU1, as indicated by the increasing band intensity of phosphorylated RegX3 and the decreasing band intensity of unphosphorylated RegX3 in the Phos-tag gel over time. When 500 μM Pi was included in the reaction mixture lacking PhoU1, SenX3SD1 phosphorylated RegX3 at a similar rate as in the reaction condition without Pi. However, the presence of PhoU1 in the reaction mixture containing 500 μM Pi resulted in a noticeable decrease in SenX3 activity in phosphorylating RegX3. Furthermore, when a more phosphorylated form of RegX3 was used in the experiment, the presence of PhoU1 in the reaction mixture containing 500 μM Pi led to the dephosphorylation of phosphorylated RegX3, whereas SenX3SD1 marginally phosphorylated RegX3 in the reaction mixture lacking PhoU1 (Fig. 7*B*). These results indicate that the presence of both PhoU1 and Pi shifts SenX3 activity toward a phosphatase-dominant state in the absence of the Pst system.

**Figure 8:**
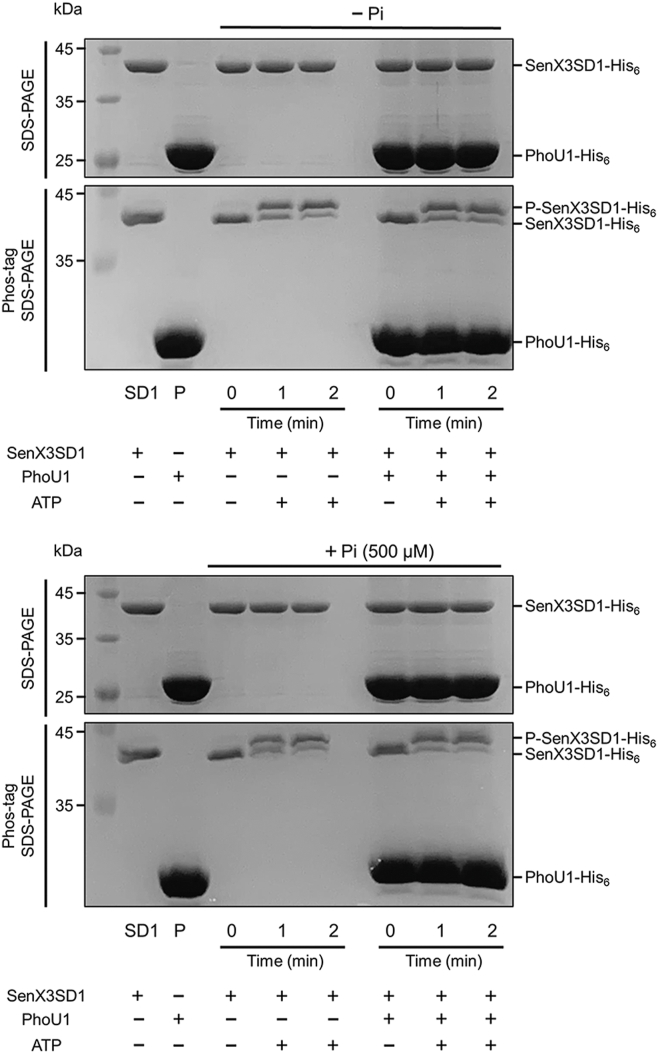
**Effects of PhoU1 and Pi on SenX3 autokinase activity.** The autophosphorylation of 46 pmol of purified SenX3SD1 was carried out in a reaction buffer [300 mM Tris-HCl (pH 8.0); 50 mM KCl; 10 mM MgCl_2_]. Where indicated, 275 pmol of purified PhoU1, 500 μM of Pi, or both were added to the reaction mixture. The preparation of the reaction mixtures was carried out on ice. Reactions were initiated by adding ATP to a final concentration of 1 mM and incubated for 2 min at 30 °C. The time point before ATP addition was designated as 0 min. Reactions were stopped by adding 3× loading buffer at the indicated time points. The reaction mixtures were then analyzed using both SDS-PAGE and Phos-tag SDS-PAGE. The gels were stained with CBB, and the bands corresponding to SenX3SD1, phosphorylated SenX3SD1 (P-SenX3SD1), and PhoU1 are indicated by *arrows*. Lanes: SD1, SenX3 SD1; P, PhoU1.

The phosphorylation of a RR by its cognate HK occurs in two steps: (i) autophosphorylation of the HK and (ii) transfer of the phosphoryl group from the autophosphorylated HK to the RR. The autophosphorylation reaction is the rate-limiting step (38). We examined whether PhoU1 decreases SenX3 autokinase activity in the presence of 500 μM Pi. As shown in Figure 8, neither PhoU1 nor Pi reduced the autophosphorylation rate of SenX3, suggesting that the observed shift of SenX3 activity toward a phosphatase mode in the presence of both PhoU1 and Pi is likely due to the inhibition of the phosphotransfer reaction, an increase in SenX3 phosphatase activity, or both.

## Discussion

In this study, we provided multiple lines of evidence supporting that SenX3 activity is controlled by PhoU in response to Pi availability, without requiring the formation of a membrane-associated signaling complex with the Pst transporter system. We found that the cytoplasmic SenX3SD1, lacking its TM domain, could effectively respond to changes in Pi levels and regulate the expression of genes of the RegX3 regulon (Fig. 2). The expression of *phoA* was still induced in the Δ*pstB* mutant of *M. smegmatis* when environmental Pi levels dropped below a certain threshold between 50 and 100 μM (Fig. 3). Moreover, the overexpression of *phoU1* or *phoU2* in the Δ*pstB* mutant appeared to at least partially restore the Pi-sensing function of the SenX3-RegX3 TCS (Fig. 6). Ectopic overexpression of *pitA*, which is expected to increase intracellular Pi levels, was shown to reduce *phoA* expression in the Δ*pstB* mutant under Pi-replete conditions (Fig. 4). Most importantly, our *in vitro* kinase and phosphatase assays clearly demonstrated that, in the presence of Pi in the reaction mixture, purified PhoU1 inhibited SenX3 activity in phosphorylating RegX3 and enhanced its phosphatase activity toward phosphorylated RegX3 (Fig. 7). Taken together, these results suggest that intracellular Pi levels are directly perceived by PhoU to regulate SenX3 activity, rather than extracellular Pi levels being sensed by the Pst system of the signaling complex. Similar claims that intracellular Pi levels influence PhoR activity have been made by several research groups (31, 32, 33). They demonstrated that PhoR activity can be influenced by the expression of proteins or cellular metabolism that alter intracellular Pi levels. According to our regulatory model of the SenX3-RegX3 TCS, its activation upon the inactivation of the Pst transporter system under Pi-replete conditions does not appear to result from the failure to form Pst-PhoU-SenX3 signaling complex but is more likely a consequence of decreased intracellular Pi levels.

**Figure 9:**
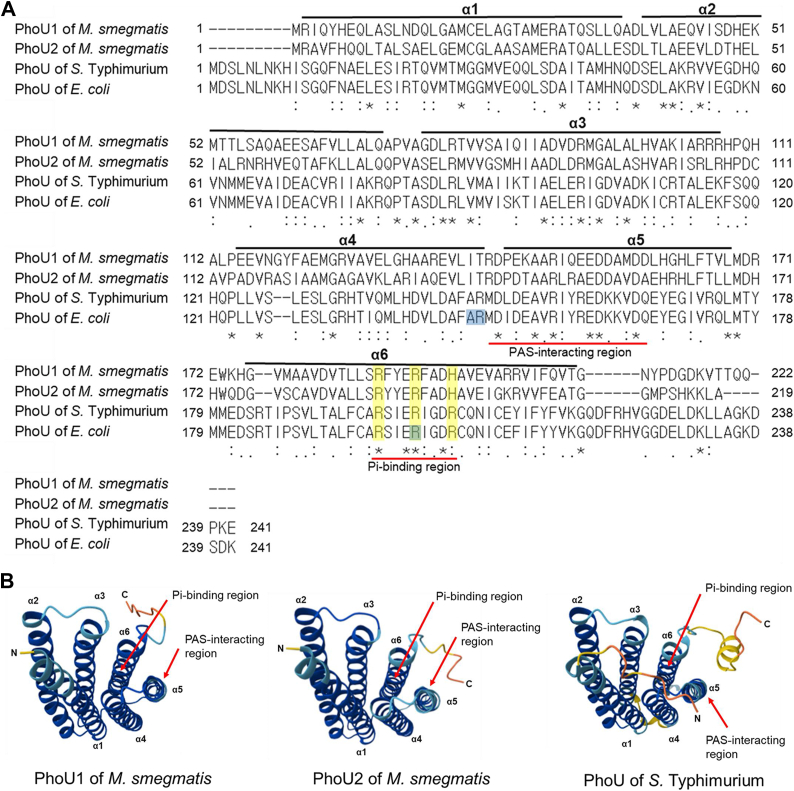
**Prediction of the Pi-binding and PAS-interacting regions on PhoU1.***A*, ClustalW-generated multiple sequence alignment of two PhoU paralogs in *M. smegmatis* and their homologs in *E. coli* and *S*. Typhimurium. The positions of the α-helices were predicted from AlphaFold-predicted structures and are indicated by *black lines* above the aligned sequences. The putative regions proposed to serve as the PAS-interacting and Pi-binding regions are marked with *red lines*. Conserved positively charged amino acids within the putative Pi-binding region are highlighted in *yellow*. The Ala147, Arg148, and Arg201 residues in *E, coli* PhoU are highlighted in *pale blue*. Identical amino acids are indicated with *asterisks*, while colons and dots denote conserved and semi-conserved substitutions, respectively. *B*, predicted three-dimensional structures of PhoU homologs generated by AlphaFold. The presented structures provide top views of the six-helix bundle. The per-residue confidence score (pLDDT) reflects prediction confidence, with 100 representing the highest confidence. The polypeptide is color-coded as follows: *orange* (pLDDT < 50), *yellow* (50 < pLDDT < 70), *light blue* (70 < pLDDT < 90), and *dark blue* (pLDDT > 90). *Arrows* indicate the positions of the putative PAS-interacting and Pi-binding regions.

The three-dimensional structures of PhoU homologs from *Pseudomonas aeruginosa*, *Thermotoga maritima*, and *Aquifex aeolicus* have been reported (39, 40, 41). All the determined structures revealed that PhoU proteins consist of two domains, each formed by two three-helix bundles. As shown in Figure 9, the AlphaFold-predicted structures of the PhoU paralogs from *M. smegmatis* and PhoU from *Salmonella* Typhimurium closely resemble the experimentally determined structures. The N-terminal domain consists of the α1, α2, and α3 helices, while the C-terminal domain is formed by the α4, α5, and α6 helices. A previous *in silico* docking model predicted that the C-terminal domain of *E. coli* PhoU binds to the cleft formed between the PAS and CA domains of PhoR. According to this model, two amino acids, Ala147 and Arg148, located at the end of the α4 helix in PhoU interact with the PAS domain of PhoR (42). The importance of Ala147 and Arg148 in PhoU’s signaling function and its interaction with PhoR was demonstrated experimentally (42). Gardner *et al.* also demonstrated that purified *E. coli* PhoU contains manganese metal ions, and that the binding of manganese or magnesium ions to PhoU induces a conformational change near the α6 helix (2). Our multiple sequence alignment of PhoU homologs revealed that two regions are relatively well conserved in the C-terminal domains of PhoU homologs (Fig. 9*A*). The first conserved region in the α5 helix is located immediately downstream of Ala147 and Arg148, leading us to hypothesize that this region may play a role in protein interactions with PhoR (SenX3). The second conserved region is located in the α6 helix and contains a conserved charged amino acid motif (RXXERXXDR/H). The presence of three positively charged amino acids (Arg or His) suggests that this motif may be involved in binding negatively charged phosphate ions, thereby playing a crucial role in the Pi-signaling function of PhoU. The previous finding that the R201 A PhoU mutant is involved in its signaling function further supports the importance of the α6 motif in Pi sensing and signaling (2).

**Figure 10:**
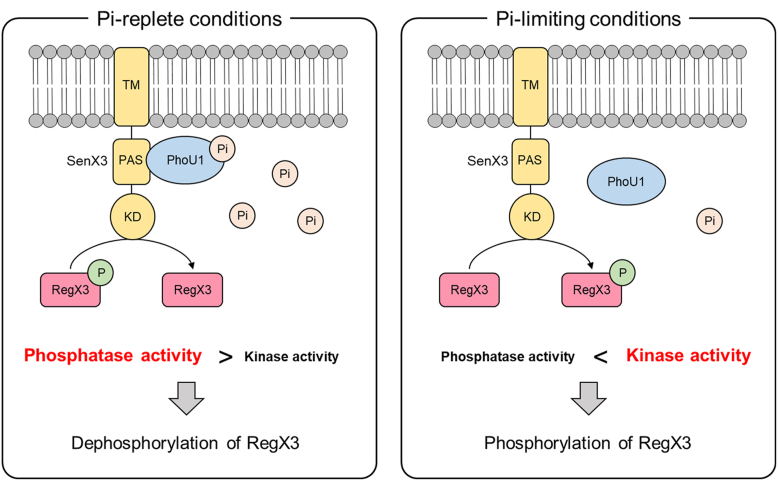
**Proposed model for Pi sensing by the SenX3-RegX3 TCS in *M. smegmatis*, based on findings from this study.** The model is explained in the Discussion section.

Based on our findings, along with previously reported results, we propose a model for Pi sensing in the SenX3-RegX3 TCS in mycobacteria (Fig. 10). The activity of SenX3 in phosphorylating the RegX3 RR is likely regulated by PhoU, with its binding affinity for SenX3 being modulated by Pi availability. Under Pi-replete conditions, Pi binding to PhoU likely at the α6 helix, induces conformational changes that are transmitted to the putative SenX3 binding region, which includes the α5 helix and the interregion between the α4 and α5 helices. In this Pi-bound form, PhoU can associate with the PAS domain of SenX3, shifting SenX3 activity toward a phosphatase-dominant mode. In contrast, under Pi-deficient conditions, PhoU exists in a Pi-unbound state, which prevents its interaction with SenX3. As a result, SenX3 remains in its default kinase-dominant state, leading to phosphorylation of the RegX3 RR.

We found that the expression of *phoU1* and *phoU2* in *M. smegmatis* was induced under Pi-deficient conditions, which is mediated by the SenX3-RegX3 TCS and SigF, respectively (Fig. 5). This upregulation likely serves to prevent the hyperactivation of the SenX3-RegX3 TCS, as excessive intracellular Pi levels, resulting from the overproduction of the Pst transporter and alkaline phosphatase, can be toxic.

In conclusion, we found that the membrane localization of SenX3 is not required for its sensory function, while its PAS domain is essential for Pi sensing. PhoU1 and PhoU2 likely sense intracellular Pi levels and regulate SenX3 kinase/phosphatase activity independently of the Pst Pi transporter. We also demonstrated that PhoU shifts SenX3 activity toward a phosphatase-dominant state in the presence of Pi, facilitating the dephosphorylation of phosphorylated RegX3. Additionally, we confirmed that PhoU1 and PhoU2 function redundantly in *M. smegmatis*. Finally, we found that the expression of *phoU1* and *phoU2* is induced under Pi-deficient conditions. The upregulation of *phoU1* is mediated by the SenX3-RegX3 TCS, while the expression of *phoU2* is regulated by SigF.

## Experimental procedures

### Bacterial strains, plasmids, and culture conditions

The bacterial strains and plasmids used in this study are listed in Table 1. *E. coli* strains were cultivated in Lysogeny Broth (LB) medium on a gyratory shaker (200 rpm) at 37 °C. *M. smegmatis* strains were grown aerobically in either Middlebrook 7H9 medium (Difco) supplemented with 0.2% (w/v) glucose (7H9-glucose) or 3-(*N*-morpholino)propanesulfonic acid (MOPS) minimal medium [25 mM MOPS (pH 7.2), 25 mM KCl, 10 mM Na_2_SO_4_, 20 mM NH_4_Cl, 10 μM FeCl_3_, 2 mM MgSO_4_, 0.1 mM CaCl_2_] supplemented with 0.2% (w/v) glucose (MOPS-glucose). To prevent clumping, 0.02% (v/v) Tween 80 was added to the *M. smegmatis* growth medium. For Pi-deficient and Pi-replete growth conditions, MOPS-glucose medium supplemented with 50 μM and 10 mM K_2_HPO_4_, respectively, was used. For growth under varying Pi concentrations, MOPS-glucose medium was supplemented with different concentrations of K_2_HPO_4_. *M. smegmatis* strains were grown aerobically on a gyratory shaker (200 rpm) to an OD_600_ of 0.45 to 0.5 at 37 °C. Antibiotics were added to the growth medium as required: ampicillin (100 μg/ml for *E. coli*), kanamycin (50 μg/ml for *E. coli* and 15 or 30 μg/ml for *M. smegmatis*), and hygromycin (200 μg/ml for *E. coli* and 50 μg/ml for *M. smegmatis*). Overexpression of a cloned gene under the control of an acetamide-inducible promoter on pMH201 derivatives was induced with 0.1% (w/v) acetamide.

**Table 1:** Bacterial strains and plasmids used in this study

Strain/plasmid	Relevant phenotype/genotype	Reference
Strains		
*E. coli* DH5α	ϕ80dl*acZ*ΔM15 Δ*lacU169 recA1 endA1 hsdR17 supE44 thi1 gyrA96 relA1*	(50)
*E. coli* BL21 (DE3)	F^-^*ompT hsd*S_*B*_ (r_B_^-^ m_B_^-^) *dcm gal* λ (DE3)	Promega
*M. smegmatis* mc^2^155	High-transformation-efficiency mutant of *M. smegmatis* ATCC 607	(44)
*M. smegmatis* Δ*sigF*	*MSMEG_1804* (*sigF*) deletion mutant of *M. smegmatis* mc^2^155	(51)
*M. smegmatis* Δ*pstB*	*MSMEG_5779 (pstB)* deletion mutant of *M. smegmatis* mc^2^155	This study
*M. smegmatis* Δ*sigB*	*MSMEG_2752* (*sigB*) deletion mutant of *M. smegmatis* mc^2^155	(34)
*M. smegmatis* Δ*senX3*	*MSMEG_0936 (senX3)* deletion mutant of *M. smegmatis* mc^2^155	This study
Plasmids		
pNC	Hyg^r^; promoterless *lacZ*	(52)
pNCII	Hyg^r^; a derivative of pNC, the ribosome-binding site of *lacZ* is removed (translational fusion)	(36)
pMH201	Km^r^; acetamide-inducible promoter, derivative of pMV306	(53)
pKOTs	Hyg^r^; pKO-based vector constructed by inserting HindIII-KpnI fragment containing pAL500 Ts and the pUC ori derived from pDE	(45)
pNBV1	Hyg^r^; 5.8-kb plasmid derived from p16R1	(54)
pT7-7	Amp^r^; T7 promoter, ribosome binding site, and translation start codon overlapping with NdeI site	(55)
pBluescript II KS +	Amp^r^; *lacZ POZ* ’	Stratagene
pNCphoA	pNC::0.485-kb XbaI-ClaI fragment containing the *phoA* promoter region	(56)
pMHSenX3WT	pMH201::1.214-kb NdeI-ClaI fragment from pT7-7SenX3WT	This study
pMHSenX3SD1	pMH201::1.074-kb NdeI-ClaI fragment from pT7-7SenX3SD1	This study
pMHphoU1	pMH201::0.692-kb NdeI-ClaI fragment containing C-terminally His_6_-tagged *MSMEG_5776* (*phoU1*)	(34)
pMHphoU2	pMH201::0.683-kb NdeI-ClaI fragment containing C-terminally His_6_-tagged *MSMEG_1605* (*phoU2*)	(34)
pMHPitA	pMH201::1.279-kb NdeI-HpaI fragment containing C-terminally His_6_-tagged *MSMEG_1064* (*pitA*)	This study
pBSSenX3	pBluescript II KS+::1.319-kb NotI-HindIII fragment containing *MSMEG_0936* (*senX3*)	This study
pBSΔsenX3	pBluescript II KS+::0.812-kb NotI-HindIII fragment containing Δ*senX3*	This study
pNBV1SenX3up	pNBV1::0.126-kb BamHI-NdeI fragment containing the upstream region of *senX3*	This study
pNBV1SenX3WT	pNBV1SenX3up::1.206-kb NdeI-HindIII fragment containing C-terminally His_6_-tagged *senX3*	This study
pNBV1SenX3SD1	pNBV1SenX3up::1.066-kb NdeI-HindIII fragment containing C-terminally His_6_-tagged, truncated *senX3*	This study
pNBV1SenX3SD2	pNBV1SenX3up::0.838-kb NdeI-HindIII fragment containing C-terminally His_6_-tagged, truncated *senX3*	This study
pNBV1SenX3SD3	pNBV1SenX3up::0.711-kb NdeI-HindIII fragment containing C-terminally His_6_-tagged, truncated *senX3*	This study
pT7-7SenX3WT	pT7-7::1.206-kb NdeI-HindIII fragment containing the gene encoding His_6_-tagged *senX3*	This study
pT7-7SenX3SD1	pT7-7::1.066-kb NdeI-HindIII fragment containing C-terminally His_6_-tagged, truncated *senX3*	This study
pT7-7RegX3	pT7-7::0.711-kb NdeI-PstI fragment containing C-termainally His_6_-tagged *MSMEG_0937* (*regX3*)	This study
pT7-7PhoU1	pT7-7::0.693-kb NdeI-HindIII fragment containing C-terminally His_6_-tagged *phoU1*	This study
pKOTsΔsenX3	pKOTs::0.812-kb NotI-HindIII fragment from pBSΔsenX3	This study
pKOTsΔpstB	pKOTs::0.812-kb HindIII fragment containing Δ*pstB*	This study
pNCⅡphoU1	pNCII::0.443-kb XbaI-ClaI fragment containing the *phoU1* promoter region	This study
pNCⅡphoU2	pNCII::0.441-kb XbaI-ClaI fragment containing the *phoU2* promoter region	This study
pNCⅡ1777	pNCII::0.456-kb XbaI-BamHI fragment containing the *MSMEG_1777* promoter region	(36)

Amp^r^, ampicillin resistance; Hyg^r^, hygromycin resistance; Km^r^, kanamycin resistance.

### DNA manipulation, transformation, and primers

Standard protocols and manufacturers’ instructions were followed for recombinant DNA manipulations (43). Transformation of *M. smegmatis* with plasmids was conducted by electroporation as previously described (44). The primers used in this study are listed in Table 2.

**Table 2:** Oligonucleotides used in this study

Oligonucleotide	Nucleotide sequence (5' → 3′)	Purpose
RT_sigA_F	CTGGAGGCGAACCTGCGC	RT-PCR for *sigA*
RT_sigA_R	CTGGTCGGCCATGGCGCG	RT-PCR for *sigA*
RT_phoA_F	GTCATCCTGCTGGTCGGC	RT-PCR for *phoA*
RT_phoA_R	GAAACCCTGTGCCTTGGC	RT-PCR for *phoA*
RT_pstS_F	GCCAGGTGTGCGGGGGCAAG	RT-PCR for *pstS*
RT_pstS_R	CCAGCTTGTCGACGCCCTCG	RT-PCR for *pstS*
F_phoU1lacZ_XbaⅠ	ATATTCTAGAGGTTTTCAACGTGACCACCCCG	pNCIIphoU1
R_phoU1lacZ_ClaⅠ	ATATATCGATGAGCTGGTCGTTCAGTGACGC	pNCIIphoU1
F_phoU2lacZ_XbaⅠ	ATATCTAGACGTCATCGACCCCTACCAGC	pNCIIphoU2
R_phoU2lacZ_ClaⅠ	ATATATCGATACCGCACATCTCGCCCAG	pNCIIphoU2
FsenX3com_BamHI	ATTAGGATCCAGGCGGTATCGGACGGG	pNBV1SenX3up
RsenX3com_NdeI	ATTACATATGAAAATCGTACGCATCCGG	pNBV1SenX3up
F_SenX3WT	ATATCATATGAGCTTGGTGTCGGCGTTAC	pNBV1SenX3WT, pT7-7SenX3WT
F_SenX3SD1	ATATCATATGCTGCAGCACATCACGTCC	pNBV1SenX3SD1, pT7-7SenX3SD1
F_SenX3SD2	ATATCATATGCGCGGCAAGGTGCGGC	pNBV1SenX3SD2
F_SenX3SD3	ATATCATATGCACGCACGCATGGAAGCCACC	pNBV1SenX3SD3
R_SenX3_his	ATATAAGCTTTCAGTGATGGTGATGGTGATGTCGTTCTCGTTGGTCCTCTCG	pNBV1SenX3 variants, pT7-7SenX3WT, pT7-7SenX3SD1
F_RegX3	AGCTCATATGACCAGCGTGTTGATCGTTGAAG	pT7-7RegX3
R_RegX3_his	AGCTCTGCAGTCAGTGATGGTGATGGTGATGGCCCTCCAGCTTGTATCCC	pT7-7RegX3
F_PhoU1	ATTACATATGCGGATCCAGTACCATGAACAG	pT7-7PhoU1
R_PhoU1_his	ATTAAAGCTTTCAGTGATGGTGATGGTGATGCTGCTGCGTGGTGACCTTGTC	pT7-7PhoU1
F_senX3_NotI	AATTGCGGCCGCCGTCGCCGCCCAGGGCGC	pKOTsΔsenX3
R_senX3_HindIII	CCGGAAGCTTTGGCGGCCACGTGTTTGAC	pKOTsΔsenX3
F_pstB_mut	ATATAAGCTTGCTGTCGGGCATCGTCACC	pKOTsΔpstB
R_pstB_rec	TCTCGGTGTCGTCGATCTCGATCCACCGTGGACTTGCCGCAG	pKOTsΔpstB
F_pstB_rec	CTGCGGCAAGTCCACGGTGGATCGAGATCGACGACACCGAGAAG	pKOTsΔpstB
R_pstB_mut	GCTACGTGCAGGTCGGGTCC	pKOTsΔpstB
F_pitAover	ATATCATATGAGCGCAGAGCTTGTCG	pMHPitA
R_pitAover	ATATGTTAACTCAGTGATGGTGATGGTGATGGACACCCGCGGGGGTGGG	pMHPitA

### Construction of plasmids

#### pKOTsΔsenX3 and pKOTsΔpstB

Temperature-sensitive suicide plasmids were used to construct mutant strains of *M. smegmatis.* To construct pKOTsΔsenX3, a PCR reaction was performed using the primers F_senX3_NotI and R_senX3_HindIII, with *M. smegmatis* chromosomal DNA as a template. The resulting 1330-bp DNA fragment, which includes a 286-bp region upstream of *senX3*, was digested with NotI and HindIII. The restricted PCR product was cloned into pBluescript II KS+, resulting in pBSsenX3. A 507-bp DNA fragment within *senX3* was deleted from pBSsenX3 using PstI. The linearized plasmid was then self-ligated at the PstI site, yielding pBSΔsenX3. pBSΔsenX3 was digested with NotI and HindIII, and the resulting 812-bp NotI-HindIII fragment was cloned into pKOTs, producing pKOTsΔsenX3.

To construct pKOTsΔpstB, we performed two rounds of recombination PCR. Using *M. smegmatis* chromosomal DNA as a template, two primary PCR reactions were conducted. The first reaction used the primers F_pstB_mut and R_pstB_rec, while the second used the primers F_pstB_rec and R_pstB_mut. These reactions generated two overlapping DNA fragments of 471 bp and 388 bp, each containing a 42-bp overlap. Both PCR products contained the same 554-bp deletion within *pstB* in their overlapping regions. In the secondary PCR, an 817-bp DNA fragment with a deletion in the *pstB* gene was amplified using the primary PCR products as templates, along with the primers F_pstB_mut and R_pstB_mut. The resulting secondary PCR product was restricted with HindIII and subsequently cloned into pKOTs digested with HindIII and EcoRⅤ, resulting in pKOTsΔpstB.

#### pNCIIphoU1 and pNCIIphoU2

pNCIIphoU1 is a *phoU1*::*lacZ* translational fusion plasmid containing the 5′ portion (58 bp) of *phoU1* and a 395-bp DNA sequence upstream of *phoU1.* To construct pNCIIphoU1, a 453-bp DNA fragment was amplified using *M. smegmatis* chromosomal DNA as a template, along with the primers F_phoU1lacZ_XbaI and R_phoU1lacZ_ClaI. The PCR product was digested with XbaI and ClaI and then cloned into the promoterless *lacZ* vector pNCII, generating pNCIIphoU1.

pNCIIphoU2 is a *phoU2*::*lacZ* translational fusion plasmid containing the 5′ portion (73 bp) of *phoU2* and a 378-bp DNA sequence upstream of *phoU2*. To construct pNCIIphoU2, a 451-bp DNA fragment was amplified using *M. smegmatis* chromosomal DNA as a template, along with the primers F_phoU2lacZ_XbaI and R_phoU2lacZ_ClaI. The PCR product was digested with XbaI and ClaI and then cloned into pNCII, resulting in pNCIIphoU2.

#### pNBV1SenX3up, pNBV1SenX3WT, pNBV1SenX3SD1, pNBV1SenX3SD2, and pNBV1SenX3SD3

pNBVSenX3up is a pNBV1-derived plasmid that contains the upstream region of *senX3*, including its promoter and ribosome-binding sequences. Using *M. smegmatis* chromosomal DNA as a template, a 131-bp fragment containing the upstream region of *senX3* was amplified by PCR with the primers FsenX3com_BamHI and RsenX3com_NdeI. The PCR product was digested with BamHI and subsequently cloned into pNBV1, which had been digested with BamHI and EcoRV, resulting in pNBV1SenX3up. DNA fragments of 1216 bp, 1076 bp, 848 bp, 699 bp, and 621 bp, which encode the C-terminally His_6_-tagged SenX3WT (amino acids 1–384), SenX3SD1 (amino acids 51–384), SenX3SD2 (amino acids 127–384), and SenX3SD3 (amino acids 151–384), respectively, were amplified by PCR. The primers used were F_SenX3WT and R_SenX3_his for SenX3WT, F_SenX3SD1 and R_SenX3_his for SenX3SD1, F_SenX3SD2 and R_SenX3_his for SenX3SD2, and F_SenX3SD3 and R_SenX3_his for SenX3SD3. The PCR products were digested with NdeI and HindIII, then cloned into pNBV1SenX3up, resulting in pNBV1SenX3WT, pNBV1SD1, pNBV1SD2, and pNBV1SD3.

#### pT7-7SenX3WT, pT7-7SenX3SD1, pT7-7RegX3, and pT7-7PhoU1

The genes encoding SenX3WT, SenX3SD1, RegX3, and PhoU1 were cloned into the pT7-7 vector to overexpress the cloned genes. To construct pT7-7SenX3WT, a 1216-bp DNA fragment encoding the C-terminally His_6_-tagged SenX3 was amplified by PCR with the primers F_SenX3WT and R_SenX3_his, using *M. smegmatis* chromosomal DNA as a template. The PCR product was digested with NdeI and HindIII and cloned into pT7-7, yielding pT7-7SenX3WT. To construct pT7-7SenX3SD1, a 1076-bp DNA fragment encoding the C-terminally His_6_-tagged SenX3SD1 was amplified by PCR with the primers F_SenX3SD1 and R_SenX3_his, using *M. smegmatis* chromosomal DNA as a template. The PCR product was digested with NdeI and HindIII and cloned into pT7-7, resulting in pT7-7SenX3SD1. For the construction of pT7-7RegX3, a 722-bp DNA fragment encoding the C-terminally His_6_-tagged RegX3 was amplified by PCR with the primers F_RegX3 and R_RegX3_his, using *M. smegmatis* chromosomal DNA as a template. The PCR product was restricted to NdeI and PstI and cloned into pT7-7, generating pT7-7RegX3. To construct pT7-7PhoU1, a 704-bp DNA fragment encoding the C-terminally His_6_-tagged PhoU1 was amplified by PCR with the primers F_PhoU1 and R_PhoU1_his, using *M. smegmatis* chromosomal DNA as a template. The PCR product was digested with NdeI and HindIII and cloned into pT7-7, resulting in pT7-7PhoU1.

#### pMHSenX3WT, pMHSenX3SD1, and pMHPitA

To construct pMHSenX3WT and pMHSenX3SD1, the pT7-7SenX3WT and pT7-7SenX3SD1 plasmids were digested with NdeI and ClaI, and the resulting DNA fragments were cloned into the pMH201 integration vector, yielding pMHSenX3WT and pMHSenX3SD1. To construct pMHPitA, a 1292-bp DNA fragment encompassing the *pitA* gene, with six His codons immediately before its stop codon was amplified by PCR using the primers F_ pitAover and R_ pitAover, with *M. smegmatis* chromosomal DNA as a template. The PCR product was restricted with NdeI and HpaI and then cloned into pMH201, resulting in pMHPitA.

### Construction of mutant strains of *M. smegmatis*

The Δ*senX3* and Δ*pstB* mutants of *M. smegmatis* were generated through allelic exchange mutagenesis using the corresponding pKOTs-derived suicide plasmids pKOTsΔsenX3 and pKOTsΔpstB, respectively. Mutagenesis was performed in the background of the WT strain, following the procedure previously described (45). In brief, the temperature-sensitive suicide plasmid was introduced into *M. smegmatis* by electroporation. Transformants were selected at 30 °C (replication-permissive temperature) on 7H9-glucose agar plates containing hygromycin. The selected transformants were then grown in 7H9-glucose liquid medium supplemented with hygromycin for 3 days at 30 °C. Heterogenotes of *M. smegmatis*, which were generated by a single recombination event, were selected for their hygromycin resistance on 7H9-glucose agar plates at 42 °C (replication-nonpermissive temperature). The selected heterogenotes were subsequently grown on 7H9-glucose liquid medium without antibiotics for 3 days at 37 °C. Isogenic homogenotes were obtained from the heterogenotes after a second recombination by selecting them for sucrose resistance on 7H9-glucose agar plates containing 10% (w/v) sucrose at 37 °C.

### **β**-Galactosidase assay and determination of the protein concentration

Cells of *M. smegmatis* were harvested, resuspended in β-galactosidase assay buffer [50 mM potassium phosphate buffer (pH 7.0), 10 mM KCl, 1 mM MgSO_4_, and 20 mM β-mercaptoethanol], and lysed using a French press. Cell-free crude extracts were obtained by centrifugation at 20,000*g* for 10 min at 4 °C. β-Galactosidase activity was assayed spectrophotometrically following a previously described procedure (46). Protein concentration was determined using a Bio-Rad protein assay kit (Bio-Rad), with bovine serum albumin serving as the standard.

### Alkaline phosphatase assay

Forty ml of aerobically grown *M. smegmatis* cultures were centrifuged at 3000×*g* for 10 min. The harvested cells were resuspended in 20 ml of ice-chilled 1 M Tris-HCl buffer (pH 8.0) containing 0.1% (v/v) Tween 80, and the OD_600_ value of the resuspended cells was determined. A 150 μl volume of the resuspended cells was added to the assay buffer [1 ml of 1 M Tris-HCl (pH 8.0) with 4 mM *p*-nitrophenyl phosphate (pNPP)], and the mixtures were incubated in the dark for 40 min at 37 °C. To stop the reaction, 100 μl of 1 M K_2_HPO_4_ was added to the mixtures. The bacterial cells were removed by centrifugation, and 1 ml of the supernatant was transferred to a cuvette. The absorbance of the supernatant was measured at 405 nm, using the assay buffer as a blank. The activity of alkaline phosphatase was calculated using the formula: A_405_/[reaction time (h) × OD_600_ of resuspended cells].

### Protein purification

#### SenX3SD1

The C-terminally His_6_-tagged truncated form of SenX3 was expressed in *E. coli* BL21 (DE3) strain harboring pT7-7SenX3SD1. The strain was cultivated aerobically to an OD_600_ of 0.4 to 0.6 in LB medium containing 100 μg/ml ampicillin at 37 °C. Expression of SenX3SD1 was induced by adding isopropyl-β-D-thiogalactopyranoside (IPTG) to a final concentration of 0.5 mM, followed by incubation for an additional 4 h at 30 °C. After harvesting 200 ml of *E. coli* culture, the cells were resuspended in 10 ml of buffer A [20 mM Tris-HCl (pH 8.0), 100 mM NaCl] containing 10 U/ml DNase I, 10 mM MgCl_2_, and 20 mM β-mercaptoethanol. The resuspended cells were disrupted twice using a French press, and cell-free crude extracts were obtained by centrifugation twice at 27,000*g* for 15 min at 4 °C. A total of 300 μl of 80% (v/v) slurry (bed volume: 240 μl) of Ni-Sepharose high performance resin (GE Healthcare) was packed into a column. After equilibrating the resin with 10 bed volumes of buffer A, the cell-free crude extracts were loaded onto the column. The resin was sequentially washed with: 60 bed volumes of buffer A containing 5 mM imidazole and 20 mM β-mercaptoethanol, 60 bed volumes of buffer A containing 25 mM imidazole and 20 mM β-mercaptoethanol, and 40 bed volumes of buffer A containing 50 mM imidazole and 20 mM β-mercaptoethanol. Finally, His_6_-tagged SenX3SD1 was eluted with 10 bed volumes of buffer A containing 250 mM imidazole and 20 mM β-mercaptoethanol.

#### PhoU1

Overexpression in *E. coli* BL21 carrying pT7-7PhoU1 and purification of the C-terminally His_6_-tagged PhoU1 were conducted following the same procedure as for SenX3SD1.

#### RegX3

Overexpression of the C-terminally His_6_-tagged RegX3 was performed in *E. coli* BL21 carrying pT7-7RegX3. The strain was cultivated aerobically in LB medium containing 100 μg/ml ampicillin at 37°C until reaching an OD_600_ of 0.4 (mid-exponential phase) or 1 (late-exponential phase). After harvesting 1 L of *E. coli* culture, the cells were resuspended in 40 ml of buffer A containing 10 U/ml DNase I, 10 mM MgCl_2_, and 20 mM β-mercaptoethanol and then disrupted using a French press. After centrifugation, imidazole was added to the separated crude extract to a final concentration of 5 mM. Subsequently, 300 μl of Ni-sepharose resin was added, and the mixture was incubated for 2 h at 4 °C with gentle shaking. The resin was washed sequentially with 30 bed volumes of buffer A containing 5 mM imidazole and 20 mM β-mercaptoethanol, 30 bed volumes of buffer A containing 10 mM imidazole and 20 mM β-mercaptoethanol, and 20 bed volumes of buffer A containing 50 mM imidazole and 20 mM β-mercaptoethanol. Finally, His_6_-tagged RegX3 was eluted with 10 bed volumes of buffer A containing 250 mM imidazole and 20 mM β-mercaptoethanol.

All purified proteins were subjected to gel-filtration chromatography using a PD-10 desalting column (GE Healthcare) or dialyzed overnight at 4 °C in 2 L of 20 mM Tris-HCl (pH 8.0) to remove NaCl and imidazole.

### Determination of protein phosphorylation using Phos-tag SDS-PAGE

#### Phosphorylation and dephosphorylation of RegX3 by SenX3SD1

Phosphorylation and dephosphorylation reactions were performed in reaction mixtures containing 300 mM Tris-Cl (pH 8.0), 50 mM KCl, and 10 mM MgCl_2_, along with the specified amounts of SenX3SD1 and RegX3. The reaction mixtures were prepared on ice. Reactions were initiated by adding ATP to a final concentration of 1 mM and incubated at 30 °C. The reactions were terminated at various time intervals by adding 3× gel-loading buffer [195 mM Tris-Cl (pH 6.8), 30% (w/v) glycerol, 3% (w/v) SDS, 15% (v/v) β-mercaptoethanol, and 0.1% (w/v) bromophenol blue]. To assess the effect of PhoU1 on RegX3 phosphorylation and dephosphorylation by SenX3SD1, reactions were performed in the presence and absence of PhoU1. To determine the effect of Pi on RegX3 phosphorylation and dephosphorylation by SenX3SD1, reactions were performed in the presence and absence of 500 μM Pi. After reaction termination, the samples were not boiled prior to PAGE but were kept at room temperature for 20 min to prevent the hydrolysis of phospho-Asp. RegX3 phosphorylation was analyzed by Phos-tag SDS-PAGE using a 12.5% (w/w) acrylamide gel containing 50 μM Mn^2+^-Phos-tag [50 μM Phos-tag acrylamide (NARD Institute Ltd, Amagasaki, Japan) and 100 μM MnCl_2_]. Gels were stained with Coomassie Brilliant Blue (CBB).

#### Autophosphorylation of SenX3SD1

The autophosphorylation reaction of SenX3SD1 was performed using the same procedure as the phosphorylation of RegX3 by SenX3SD1, except that RegX3 is excluded from the reaction mixture. SenX3SD1 phosphorylation was analyzed by Phos-tag SDS-PAGE using an 8% (w/w) acrylamide gel containing 75 μM Mn^2+^-Phos-tag [75 μM Phos-tag acrylamide and 150 μM MnCl_2_].

### Western blot analysis

Cell-free crude extracts were subjected to SDS-PAGE, and proteins on the gel were transferred onto polyvinylidene fluoride membranes (Millipore). Western blotting was performed as described previously (47). To detect His_6_-tagged proteins, a mouse monoclonal IgG against His_6_ (Thermo Fisher Scientific; MA1-21315) was used at a 1:2000 dilution. GroEL was detected using a mouse monoclonal antibody against Hsp65 (Santa Cruz Biotechnology; sc58170) at a 1:2000 dilution. A horseradish peroxidase-conjugated anti-mouse IgG (Bio-Rad) was applied at a 1:10,000 for the detection of the primary antibodies. Protein bands were visualized using an ECL kit (Advansta) and imaged with a ChemiDoc system (Bio-Rad).

### Quantitative real-time PCR (qRT-PCR)

RNA isolation from *M. smegmatis* strains and cDNA synthesis were performed as previously described (48), except that a random hexamer primer (Thermo Fisher Scientific) was used instead of gene-specific primers in cDNA synthesis. The isolated RNA was assessed for DNA contamination by PCR using the primers designed for qRT-PCR. qRT-PCR was performed to determine the transcript levels of *phoA*, *pstS*, and *sigA*. The reaction was conducted in a 20-μl mixture containing 5 μl of template cDNA, 1 μl (15 pmol) of each gene-specific primer, 10 μl of TB Green Premix Ex Taq (Tli RNase Plus) (Takara), 0.4 μl of ROX passive fluorescent dye, and 2.6 μl of distilled water. The thermal cycling program included an initial denaturation step at 95 °C for 2 min, followed by 40 cycles of 95 °C for 5 s and 64 °C for 30 s. The *sigA* gene, encoding the principal sigma factor, was used as a reference gene in qRT-PCR to normalize the expression levels of *phoA* and *pstS*. Melting curve analysis was performed for each reaction to confirm the amplification of a single PCR product.

### Statistical analysis

Statistical analysis was performed using GraphPad Prism 10.0 (GraphPad Software Inc). Each experiment included at least three biological replicates. Data were analyzed using an unpaired Student’s *t* test, and differences were considered statistically significant at *p* < 0.05.

## Data availability

The transcriptomic data used in the Result section were obtained from the NCBI GEO database using the accession number GSE155251.

## Conflict of interest

The authors declare that they have no conflicts of interest with the contents of this article.
